# Radiology Gets Chatty: The ChatGPT Saga Unfolds

**DOI:** 10.7759/cureus.40135

**Published:** 2023-06-08

**Authors:** Harpreet Grewal, Gagandeep Dhillon, Varun Monga, Pranjal Sharma, Venkata S Buddhavarapu, Gurmanpreet Sidhu, Rahul Kashyap

**Affiliations:** 1 Radiology, Florida State University College of Medicine, Pensacola, USA; 2 Internal Medicine, Baltimore Washington Medical Center, Glen Burnie, USA; 3 Psychiatry, Banner Health, Phoenix, USA; 4 Nephrology, Northeast Ohio Medical University, Rootstown, USA; 5 Hospital Medicine, Banner Health, Phoenix, USA; 6 Pathology, Government Medical College, Patiala, Patiala, IND; 7 Medicine, Drexel University College of Medicine, Philadelphia, USA; 8 Global Clinical Scholars Research Training, Harvard Medical School, Boston, USA; 9 Research, Global Remote Research Scholars Program, Saint Paul, USA; 10 Critical Care Medicine, Mayo Clinic, Rochester, USA

**Keywords:** openai, ai chatbot, language learning model, general radiology, chat gpt, artificial intelligence in radiology

## Abstract

As artificial intelligence (AI) continues to evolve and mature, it is increasingly finding applications in the field of healthcare, particularly in specialties like radiology that are data-heavy and image-focused. Language learning models (LLMs) such as OpenAI's Generative Pre-trained Transformer-4 (GPT-4) are new in the field of medicine and there is a paucity of literature regarding the possible utilities of GPT-4 given its novelty. We aim to present an in-depth exploration of the role of GPT-4, an advanced language model, in radiology. Giving the GPT-4 model prompts for generating reports, template generation, enhancing clinical decision-making, and suggesting captivating titles for research articles, patient communication, and education, can occasionally be quite generic, and at times, it may present factually incorrect content, which could lead to errors. The responses were then analyzed in detail regarding their potential utility in day-to-day radiologist workflow, patient education, and research processes. Further research is required to evaluate LLMs' accuracy and safety in clinical practice and to develop comprehensive guidelines for their implementation.

## Introduction

Radiology is an important component of modern healthcare, providing clinicians with essential information for accurate diagnosis and management of various medical conditions. Recent advances in artificial intelligence (AI) and machine learning (ML) have led to the development of innovative tools that can enhance the diagnostic capabilities of radiologists.

ChatGPT (Chat Generative Pre-training Transformer), an advanced language learning model (LLM) developed by OpenAI (San Francisco, California, United States) [1], is trained on a massive dataset of text and code and can generate text, translate languages, write different kinds of creative content, and answer questions in an informative way [2]. It has shown promising utility for radiologists to improve diagnostic efficiency, increase the accuracy of report translation in various languages, and the breaking down terminology into easier-to-understand language [3]. We aim to discuss the role of ChatGPT in radiology, outlining the applications (with the help of relevant examples of human prompts and AI responses), with a balanced and comprehensive insight into ChatGPT's applications, limitations, and ethical considerations in radiology.

## Materials and methods

This is a cross-sectional technology validation study. The study utilizes real-time prompting and response evaluation from the LLM, GPT-4 (Generative Pre-trained Transformer-4). No real patient data or variables are used in this study. The primary objective was to demonstrate the role of ChatGPT in radiology, outlining the applications with relevant examples of human prompts and AI responses. 

Study procedure

For this study, we used OpenAI's GPT-4, the latest version of the model available at the time of writing this article in the first week of May 2023.

Prompting

Specifically tailored prompts were provided to the GPT-4 model for generating reports, template generation, enhancing clinical decision-making, suggesting captivating titles for research articles, and patient communication and education. One follow-up prompt was used in enhancing clinical decision-making and generating captivating titles for research article sections each. Otherwise, no manipulation or refinement of the prompts was performed during the study.

Validation

The responses were captured as is, without alteration. The responses were then analyzed in detail in regard to their potential utility in day-to-day radiologist workflow, patient education, and use in research processes by an expert radiologist and co-author (HG).

Information Analyses

The information was subsequently analyzed for context and content that is not only applicable to providers but also useful to the patient.

Ethical considerations

Since the study focuses on utilizing real-time indications and evaluating responses from the LLM, GPT-4 AI tool, without using data, biological samples, or patient participation, for purposes such as generating reports, creating templates, enhancing clinical decision-making, suggesting captivating titles for research articles, and patient communication and education, it does not require validation by ethics committees in accordance with the Helsinki Declaration.

## Results

Here, we present several prompts and responses from GPT-4 and clinicians’ analysis on these. Also, we have summarized them as applications of ChatGPT in Radiology.

Generating reports

ChatGPT can automatically generate simpler or less-complicated radiology reports based on conventional imaging findings by conducting extensive searches of existing literature related to radiology, significantly cutting down on radiologists' report writing time. These automated reports have demonstrated a high degree of accuracy, consistency, and clinical relevance [4]. However, the practicality of these generated reports may be questionable and appears more as an unusual novelty, given that current radiology dictation software utilizes various voice commands to produce pre-stored templates quickly for reporting [5]. An example of human prompting and ChatGPTs response is:

Human prompt: There is a 4 cm hepatic hemangioma in segment VI of liver. Please write an MRI abdomen report on this. Also add follow-up recommendation in the impression.

ChatGPT response: Sure, here's an example of an MRI Abdomen report for a 4 cm hepatic hemangioma in segment 6 of the liver:

FINDINGS:

· Liver: A 4 cm hepatic hemangioma is identified in segment VI of the liver. The lesion appears hyperintense on T2-weighted images with a central area of low signal intensity ("flow void"), and has low to intermediate signal intensity on T1-weighted images. On contrast-enhanced MRI, the lesion shows peripheral enhancement in a nodular or rim-like pattern, with delayed central enhancement due to the slow flow of blood within the lesion.

· Gallbladder: No significant abnormality.

· Pancreas: Normal T1 hyperintense signal. No intrapancreatic lesion identified. Main pancreatic duct not dilated. No peripancreatic fluid collection identified.

· Spleen: Normal in size. No splenic lesion.

· Kidneys: Normal signal intensity. No hydronephrosis. No discrete renal lesion.

· Bowel: No bowel obstruction. Normal caliber.

· Osseous structures: No acute osseous abnormality.

· Lymph nodes: No lymphadenopathy.

· Adrenal glands: Grossly within normal limits.

· Vessels: Normal caliber abdominal aorta.

IMPRESSION:

1. Four cm hepatic hemangioma identified in segment VI of the liver, consistent with the imaging characteristics of a hepatic hemangioma.

2. No other significant abnormality was identified.

FOLLOW-UP RECOMMENDATION: Given the size of the hepatic hemangioma, follow-up imaging in 6-12 months is recommended to assess for any interval changes in size or imaging characteristics. Close clinical monitoring and further management recommendations should be based on the results of the follow-up imaging.”

Template generation

Template generation in radiology aims at producing structured reports from free text descriptions in an effort to streamline report production more quickly and effectively. ChatGPT leverages natural language processing (NLP) techniques to extract relevant data and organize it according to predefined templates. This may include patient demographics, clinical history, imaging modality used, anatomical structures examined, or pathologic findings. Radiology reports are generated using free text by extracting information corresponding to sections of the template, populating said template with relevant data, and making sure language and format meet standard radiology reporting guidelines and terminology [6]. Below is an example response generated by Chat GPT after being given instructions to create a CT lung cancer screening protocol.

*Human prompt: Can you please generate a template for CT lung cancer screening. Also add follow-up recommendations based on the *Lung CT Screening Reporting & Data System (Lung-RADS^®^)* categories.*

ChatGPT response: Sure, here's a template for CT lung cancer screening that includes follow-up recommendations based on the Lung-RADS categories:

TECHNIQUE: Non-contrast CT scan of the chest was performed with the following parameters: __________.

FINDINGS:

• Lungs: The lung parenchyma is clear without any focal nodular opacities or masses. No evidence of emphysema or bronchiectasis. No pleural or pericardial effusions.

• Mediastinum: The mediastinum is normal in appearance. No lymphadenopathy or masses identified.

• Heart: The heart is normal in size and unremarkable.

• Other structures: No significant abnormality identified.

IMPRESSION: The CT scan of the chest is negative for any evidence of lung cancer or other significant abnormalities. However, this is a screening examination and follow-up may be necessary based on the patient's risk factors and clinical presentation. Based on the patient's risk factors and imaging findings, the following follow-up recommendations are suggested according to the Lung-RADS:

Lung-RADS Category 1: Negative

• Routine screening recommended based on age and smoking history.

Lung-RADS Category 2: Benign

• Routine screening recommended based on age and smoking history.

Lung-RADS Category 3: Probably Benign

• Annual screening recommended for 2 years, then routine screening based on age and smoking history.

Lung-RADS Category 4A: Suspicious

• Immediate diagnostic workup recommended.

Lung-RADS Category 4B: Highly Suspicious

• Immediate diagnostic workup recommended.

Lung-RADS Category 4X: Known Biopsy-Proven Malignancy

• Post-treatment surveillance recommended as per standard practice.

Lung-RADS Category 5: Malignancy

• Appropriate management and treatment recommended as per standard practice.

Please note that these follow-up recommendations are general guidelines and may vary depending on the specific clinical context and the patient's individual risk factors and medical history.”

Patient communication and education

ChatGPT can serve as an invaluable aid for radiologists and care team providers when conveying complex information to patients in an understandable manner. Utilizing NLP capabilities, ChatGPT generates patient-oriented explanations of radiological findings as well as implications for care using tailored prompts incorporated within its NLP model to deconstruct complex medical terminology into an easily understandable narrative [6]. Here is an example of simplification by ChatGPT of an entire radiology report.

*Human prompt: I got my MRI results back today and my MRI abdomen report states ' 5 cm arterially enhancing lesion in segment VII of liver with arterial phase enhancement and washout on portal venous phase and a pseudocapsule, compatible with HCC (*Liver Reporting & Data System (LI-RADS^®^)*)'. Please simplify this report for me. *

ChatGPT response: I'm not a doctor, but I can try to help you understand the terminology in your MRI report. It seems to be describing a finding in your liver:

"5 cm arterially enhancing lesion": A lesion (abnormal tissue) measuring 5 centimeters is seen in the liver. It appears to be receiving blood supply from the arteries, which is causing it to stand out on the MRI.

"Segment VII of liver": The liver is divided into segments, and this lesion is found in the seventh segment.

"Arterial phase enhancement and washout on portal venous phase": This means the lesion appears brighter during the arterial phase (when blood is primarily supplied by the arteries) and then fades during the portal venous phase (when blood flows from the portal vein into the liver). This pattern is sometimes seen in certain types of liver lesions.

"Pseudocapsule": A pseudocapsule is a thin layer of tissue that can surround a lesion, giving it the appearance of being encapsulated.

"Compatible with HCC (L-RADS 5)": HCC stands for hepatocellular carcinoma, which is a type of liver cancer. LI-RADS is a classification system for liver lesions, with 5 being the highest category, suggesting a high likelihood of HCC.

In summary, your MRI report describes a 5 cm lesion in segment VII of your liver, with characteristics that suggest it is likely to be hepatocellular carcinoma (liver cancer). However, you should consult your doctor to discuss these findings, receive a proper diagnosis, and discuss any necessary next steps.”

ChatGPT as a conversational AI assistant for patient queries

Integrating ChatGPT as a chatbot for patient inquiries involves the creation of an engaging, user-friendly platform whereby patients may pose their inquiries and receive accurate, clear, and tailored responses regarding medical imaging results or healthcare concerns. This can be accomplished by setting out the scope, such as radiology reports, medical terminology, follow-up recommendations, and customizing and training ChatGPT using relevant datasets while designing an intuitive user experience and accessibility options. Integration with electronic health records (EHRs) that adhere to privacy regulations can create an enhanced patient experience, with constant monitoring of performance and gathering user feedback for continuous service improvement and producing accurate, up-to-date, and relevant data [7].

Enhance clinical decision-making processes

ChatGPT cannot directly interpret images yet can play an integral part in clinical decision-making by synthesizing meta-analyses, clinical trials, and research papers into summaries that add value to the decision-making process. When provided with details from an imaging modality's findings, the model provides a list of differential diagnoses for consideration.

ChatGPT can serve as an aid to radiologists conducting intelligent research [6]. Radiologists should utilize creativity when drafting prompts for ChatGPT to obtain desired information yet verify and apply one's medical knowledge in order to reduce potential errors [7]. Below is an example of how ChatGPT may assist radiologists by helping generate differentials.

Human prompt 1: The right knee radiograph of a 44-year-old male shows a lesion in the lesion in the lateral tibial plateau. The lesion is well-circumscribed with faint sclerotic margins. The lesion shows no cortical break or endosteal scalloping. I do not have significant matrix calcifications. Please tell me the differentials for this lesion.

ChatGPT response 1: I am not a radiologist or a physician, but I can try to provide you with some general information on differentials for the described lesion. Keep in mind that a definitive diagnosis should always be made by a qualified healthcare professional.

Based on the description provided, some possible differentials for a well-circumscribed lesion with faint sclerotic margins in the lateral tibial plateau of a 44-year-old male might include:

1. Bone cyst: This can be either a simple bone cyst (unicameral bone cyst) or an aneurysmal bone cyst. Both are benign bone lesions, but they have different characteristics and treatment options.

2. Enchondroma: A benign cartilage-forming tumor that can occur within the medullary cavity of the bone. It is typically asymptomatic and discovered incidentally.

3. Fibrous dysplasia: A benign bone disorder characterized by the replacement of normal bone with fibrous tissue and immature bone. It can cause pain, deformity, and fractures.

4. Non-ossifying fibroma: A benign, asymptomatic lesion composed of fibrous tissue, typically found in the metaphysis of long bones. These lesions often resolve spontaneously over time.

5. Chondrosarcoma: A malignant cartilage-forming tumor that can occur in the medullary cavity or on the surface of the bone. It is less likely in this case due to the absence of significant matrix calcifications and cortical destruction.

6. Osteoid osteoma: A small, benign bone tumor that typically causes pain, particularly at night. It is less likely in this case because it is usually accompanied by a radiolucent nidus with surrounding sclerotic margins, which is not mentioned in the description.

7. Intraosseous ganglion: A benign, cystic lesion filled with mucinous material. It is typically asymptomatic and can be found incidentally.

8. Brodie's abscess: A subacute or chronic osteomyelitis that presents as a well-circumscribed lesion with sclerotic margins. It is usually accompanied by a history of infection or trauma.

These are just a few possibilities to consider. A thorough clinical evaluation and additional imaging studies, such as MRI or CT scan, may be necessary to further evaluate the lesion and establish a definitive diagnosis. Always consult with a healthcare professional for proper evaluation and management.

Human prompt 2: Why did you not include a giant cell tumor (GCT) in your differential?

ChatGPT response 2: You are correct that a GCT could be a potential differential diagnosis for the described lesion. I apologize for not including it in my initial list.

GCTs are benign but locally aggressive bone tumors that commonly occur near the ends of long bones, often around the knee. They tend to appear as lytic, eccentric lesions with well-defined margins, sometimes with a thin rim of sclerosis. Although cortical destruction and soft tissue extension are more commonly seen in GCTs, some cases may not present with these features, making the diagnosis more challenging.

Given the variable presentation of bone lesions, it is essential to correlate the imaging findings with the patient's clinical history and other available diagnostic information. A biopsy may be necessary to confirm the diagnosis in some cases. As always, consult with a healthcare professional for proper evaluation and management.

Propose compelling and captivating titles for research publications

ChatGPT can be an effective tool to generate impactful titles for research articles. By providing essential details such as research questions and topics to its model, ChatGPT can create compelling titles which capture readers' interest while broadening the dissemination of findings. Below is an example of how ChatGPT generates titles for an article.

Human prompt 1: Suggest me some titles for a review article on the role of ChatGPT in radiology.

ChatGPT response 1:

· ChatGPT in Radiology: A Comprehensive Review of Applications and Challenges

· The Emerging Role of ChatGPT in Radiology: Opportunities and Limitations

· ChatGPT and Radiology: A New Frontier in Artificial Intelligence and Medical Imaging

· Harnessing the Power of ChatGPT in Radiology: Potential Applications and Ethical Considerations

· ChatGPT in Radiology: Transforming Clinical Decision-Making and Patient Communication

· Navigating the Role of ChatGPT in Radiology: A Review of Current Practices and Future Perspectives.

Human prompt 2: Please make these titles catchier rather than mundane.

ChatGPT response 2:

· ChatGPT Revolution: Unveiling the Future of Radiology

· The ChatGPT Phenomenon: Transforming Radiology in the AI Era

· Radiology Reimagined: Exploring the ChatGPT Effect

· ChatGPT and the Radiologist's Virtual Assistant: A Bold New Partnership

· Beyond Traditional Radiology: The Impact of ChatGPT on Medical Imaging

· The ChatGPT Chronicles: Redefining Radiology Through Artificial Intelligence

Generate engaging and impactful headings for scholarly articles

Though ChatGPT cannot entirely produce academic papers on its own, it provides invaluable assistance in organizing the manuscript and suggesting essential components for each section. When given sufficient information about a research question or hypothesis, ChatGPT offers preliminary language that could form part of an introduction section. For authors writing non-native language papers, ChatGPT can assist by editing drafts to make them grammatically correct and clearly articulated [8].

Formatting and references for research papers

Academic radiologists must ensure accurate referencing. ChatGPT can provide invaluable assistance with formatting bibliographies in accordance with various citation styles like American Psychological Association (APA), Modern Language Association (MLA), or the Chicago referencing styleto streamline this process [8]. However, the inability of ChatGPT to generate accurate references is a major limitation. Fabricated and non-existent references are also noticed while testing its functionality. Clinicians and researchers should be mindful of this significant drawback.

## Discussion

Large language learning models such as ChatGPT showed a reasonable output after giving targeted prompts. It could be a useful tool, not only as a virtual assistant [9] but also help with streamlining various tasks for radiologists and other healthcare providers [10-12].

It is important to note that the last training data for ChatGPT is from September 2021, at the time of writing this article. Also, while it can provide follow-up recommendations for medical imaging, it is possible that these recommendations may be inaccurate or may not reflect the opinion of a radiologist. As such, the follow-up recommendations generated by ChatGPT should not be entirely relied upon for medical decision-making or patient care. Radiologists must exercise their own professional judgment and base their recommendations on the specific clinical context of each patient's case.

The latest version of ChatGPT (GPT-4) is based on an extensive dataset, trained using a significant number of neural networks, which allows it to perform complex analytical tasks [13]. While ChatGPT can generate text-based reports based on input text, its clinical utility remains questionable as it cannot directly interpret medical images.

ChatGPT is indeed effective at generating a comprehensive list of differentials when provided with a description of imaging findings. However, its inability to accept and analyze actual images considerably limits its utility as an assistant for radiologists in their daily work, as of now. Furthermore, ChatGPT can sometimes appear overly agreeable, which may be potentially hazardous for novice trainees who rely on it for guidance. As such, radiologists should always be mindful of these limitations and never solely depend on it for generating differentials.

Limitations and ethical considerations

Despite the numerous benefits provided by ChatGPT in radiology, it is essential to recognize its limitations and address potential ethical concerns. As an AI tool, ChatGPT is prone to errors and biases which are inherent in the training data, potentially leading to inaccuracies. The growing reliance on AI systems raises questions regarding the role of radiologist expertise in decision-making and the implications for accountability and liability. When ChatGPT contributes to clinical decisions, it is vital to ensure that radiologists remain engaged in the process, offering oversight, and applying their medical knowledge and expertise. Establishing clear policies for ChatGPT usage and error management is also essential [8].

Integrating ChatGPT with EHRs presents challenges, primarily concerning patient privacy and potential data leakage, which would violate Health Insurance Portability and Accountability Act (HIPAA) regulations [2]. Additionally, achieving seamless coordination between ChatGPT and EHRs necessitates cooperation among multiple IT platforms, a complex task due to the numerous variables involved.

Furthermore, ChatGPT's training data is limited to information available up until September 2021 (compared to the writing of this article on May 2023) which poses a significant delay as it may not incorporate the latest advancements or updated guidelines in radiology, potentially affecting the accuracy of the generated content.

The information generated by ChatGPT can occasionally be quite generic, and at times, it may present factually incorrect content, which could lead to errors. As such, users should exercise caution when relying on AI-generated content [14]. Interestingly, it has been observed that when the chatbot is repeatedly asked similar questions, the responses can vary, creating potential confusion for users seeking to learn more about a disease process or, even more critically, the prognosis of a specific condition. These limitations present challenges for the direct implementation of chatbots in both trainee and patient education; however, they may still serve as a valuable supplementary tool when used with caution.

Future directions

Currently, ChatGPT, in its fourth iteration, lacks the capability to process image input and therefore cannot assist with image interpretation directly. Further research is required to evaluate LLMs' accuracy and safety in clinical practice and to develop comprehensive guidelines for their implementation. The field of AI-powered chatbots is constantly evolving with daily advancements, such as the recent Bard Experiment by Google (Google LLC, Mountain View, California, United States) [15]. However, determining their appropriate roles and level of autonomy presents challenges. Additionally, ambiguity around the legal liability associated with their use necessitates clear guidelines for their implementation [16].

## Conclusions

ChatGPT's role as a virtual assistant with the help of human prompts and AI responses has shown tremendous opportunities in our pilot study for streamlining various tasks for radiologists. Large language learning models such as ChatGPT possess significant potential to function as versatile virtual assistants for other healthcare providers as well. As this technology is still in its early stages, the future role of ChatGPT and AI in radiology remains to be seen. It is crucial to address the limitations and ethical considerations to ensure its safe and responsible integration into clinical practice.
